# An integrated strategy for identifying new targets and inferring the mechanism of action: taking rhein as an example

**DOI:** 10.1186/s12859-018-2346-4

**Published:** 2018-09-06

**Authors:** Hao Sun, Yiting Shen, Guangwen Luo, Yuepiao Cai, Zheng Xiang

**Affiliations:** 1 School of Pharmaceutical Sciences, Wenzhou Medical University, Wenzhou, 325035 China; 2 Pharmacy Department, Women’s Hospital, Zhejiang University School of Medicine, Hangzhou, 310006 Zhejiang China

**Keywords:** Target identification, Rhein, Ligand-protein docking, Network analysis, Enrichment analysis, SPR

## Abstract

**Background:**

Target identification is necessary for the comprehensive inference of the mechanism of action of a compound. The application of computational methods to predict the targets of bioactive compounds saves cost and time in drug research and development. Therefore, we designed an integrated strategy consisting of ligand-protein docking, network analysis, enrichment analysis, and an experimental surface plasmon resonance (SPR) method to identify and validate new targets, and then used enriched pathways to elucidate the underlying pharmacological mechanisms. Here, we used rhein, a compound with various pharmacological activities, as an example to find some of its previously unknown targets and to determine its pharmacological activity.

**Results:**

A total of nine candidate targets were discovered, including LCK, HSP90AA1, RAB5A, EGFR, CDK2, CDK6, GSK3B, p38, and JNK. LCK was confirmed through SPR experiments, and HSP90AA1, EGFR, CDK6, p38, and JNK were validated through previous reports. Rhein network regulations are complex and interconnected. The therapeutic effect of rhein is the synergistic and comprehensive result of this vast and complex network, and the perturbation of multiple targets gives rhein its various pharmacological activities.

**Conclusions:**

This study provided a new integrated strategy to identify new targets of bioactive compounds and reveal their molecular mechanisms of action.

**Electronic supplementary material:**

The online version of this article (10.1186/s12859-018-2346-4) contains supplementary material, which is available to authorized users.

## Background

In real biological systems, bioactive compounds generally bind to more than one target proteins to exert their biological activities [1]. Target identification is therefore necessary for the comprehensive inference of the action mechanisms of a compound. Although wet lab experiments are more convincing, the application of in silico computational methods to predict targets of bioactive compounds has become more important in recent years [2]. Current computational methods for drug target discovery fall into three categories: structure-based, ligand-based, and phenotype-based virtual screening [3]. The structure-based methods involve the molecular docking between a ligand and a target, and the scoring function is used to assess the likelihood of the ligand binding to a protein. The disadvantages of this method include high false positives and weak accuracies [4]. The ligand-based methods are based on using similarities between known ligands to speculate on unknown structures of receptor sites; thus, such methods are not appropriate for the analysis of proteins without known ligands [5]. The phenotype-based methods aim at analysing phenotypic responses, such as gene expression profiles in cell lines or proteomic information, but may neglect valuable information from other types of data sources [6]. Perhaps, any method used alone will have its own short board, so the combination of multiple methods is a train of thought.

Actually, an effective drug often regulate several biological processes by acting on multiple targets, which can form a complex interaction network [2]. The complex network can provide a lot of target topological information through network analysis. Therefore, the network analysis can be used to study the complex interactions between targets and may be a good method for new target identification. However, it cannot reflect the whole biological processes since how targets influence the biological processes are lacked. The enrichment analysis can link interactions between proteins and biological processes. Therefore, the enrichment analysis can supplement the deficiency of network analysis for identifying targets and inferring their regulation on biological processes [7]. Nowadays, network visualisation and bioinformatics enrichment tools have promoted the understanding of complex drug-target and target-target interactions, accelerated the drug discovery through the identification of topological structures in biological networks, developed a systematic understanding of drug action and disease complexity, and improved the efficiency and safety of drug design [8–10].

Rhein is an active alipophilic anthraquinone that is mainly extracted from several traditional plant rhizomes, including *Rheum palmatum* L., *Aloe barbadensis* Miller, *Cassia angustifolia* Vahl*.*, and *Polygonum multiflorum* Thunb. [11]. Rhein has various pharmacological effects, such as anti-inflammatory, anti-tumour, antioxidant, antifibrotic, hepatoprotective, and nephroprotective activities [12, 13]. According to our research, more than 1000 articles about rhein have been published in PubMed; over 100 of these have discussed its pharmacological mechanism of action [13]. Many targets of rhein have been identified in recent years. Rhein could suppress all the tested RXRA-involved homo-or-heterodimeric transcription activities, decrease the expression of VEGFA, EGF, HIF1A, ERBB2, and PTGS2 proteins, decrease the activity of NFKB1 and RELA proteins [14, 15], and increase the levels of apoptosis-related proteins including BAX, CASP3, and CASP8 [16]. Moreover, the regulation of multiple pathways by rhein, such as the MAPK, PI3K-AKT, NF-κB, and TGF-β signalling pathways, cell cycle, and cell apoptosis, has been a particular focus of research [17–19]. Since rhein affects so many different targets and regulates multiple pathways in the body, we believe that rhein can be repurposed to treat even more diseases, and its new targets can still be discovered.

In this study, an integrated strategy consisting of ligand-protein docking, network analysis, enrichment analysis, and experimental validation was developed and applied to identify new rhein targets and infer the mechanisms underlying the pharmacological effects of rhein. Using this approach, we could easily identify the targets of one drug or one bioactive compound and infer their molecular mechanisms.

## Methods

**Fig. 1:**
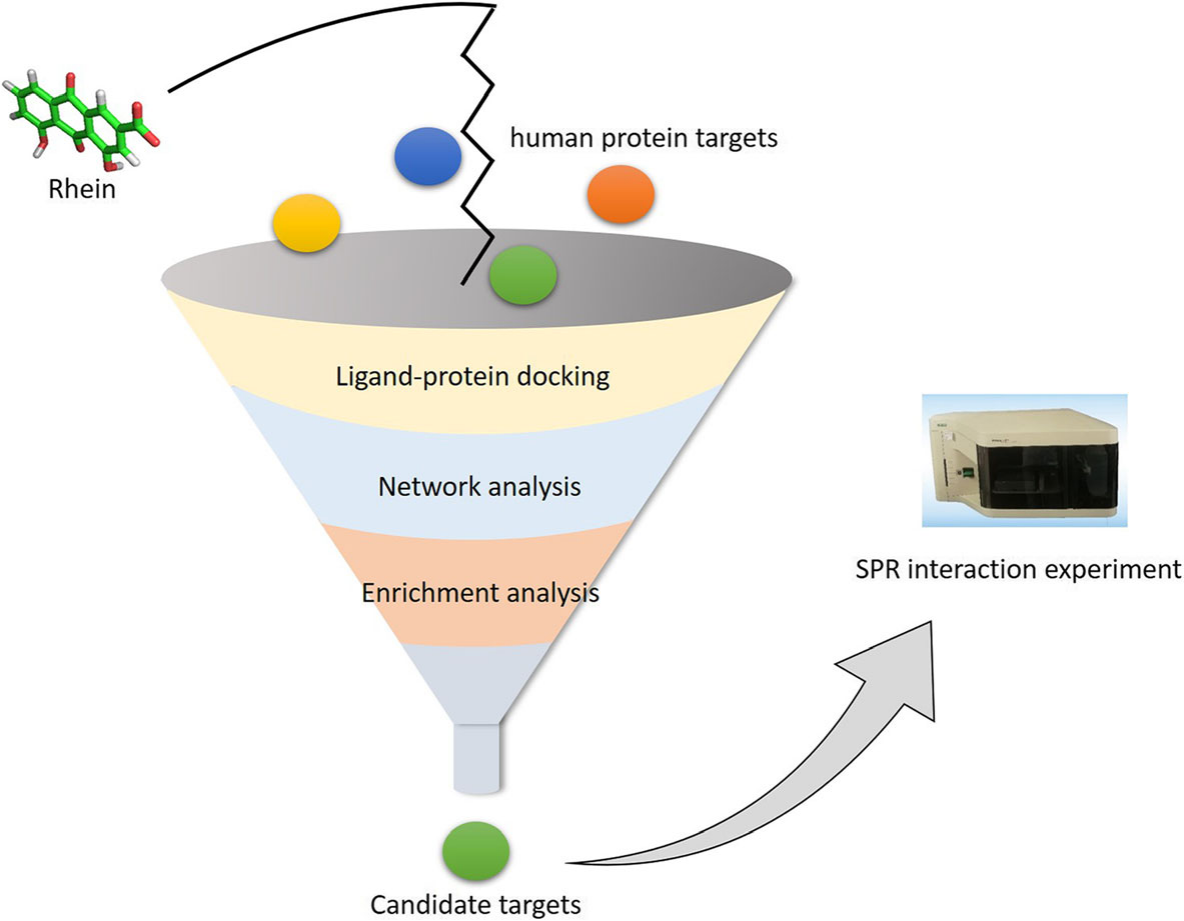
The strategy of the target identification

The integrated strategy for target identification involved four main steps: (1) Preliminary screening by ligand-protein docking; (2) Further screening by network analysis; (3) Final screening by enrichment analysis; (4) Validating candidate targets through the surface plasmon resonance (SPR) interaction experiment. The strategy of target identification is shown in Fig. 1.

### Ligand-protein docking for potential targets

Here, two steps were designed for the preliminary screening of targets. First, the inverse molecular docking, one of the ligand-based virtual screening, was used to quickly narrow the screening range of potential targets by the fit scores. Then, the accurate molecular docking, one of the structure-based virtual screening, was used to further screen potential targets.

For the inverse molecular docking analysis, the 3D molecular structure of a compound of interest (downloaded from the ZINC database [20]) was uploaded to the PharmMapper Server. The PharmMapper was a freely accessible web server designed to discover potential targets for given molecules using the pharmacophore mapping approach. It was backed up by a large pharmacophore database that includes 2241 human protein targets extracted from TargetBank, DrugBank, BindingDB, and PDTD [21]. Here, the “select targets set” parameter was set as “human protein targets only”, and all other parameters were set to their default values. Based on the fit score, the top 300 proteins (default values) were obtained and referred to as the potential targets; their 3D molecular structures were downloaded from the Protein Data Bank [22].

Due to the low screening threshold for the inverse molecular docking, accurate molecular docking is used for further screening. All the potential targets were pre-processed with PyMOL [23]. Water molecules, metal ions, and other small molecules were removed from the model. Hydrogen atoms were then added, and all non-hydrogen atoms were not allowed to move. The search space for each target was determined according to the coordinate and size of the experimental-bound ligand structure. Subsequently, all structure files of the pre-processed targets and their experimental ligands were saved. To obtain the most stable conformations, all experimental ligands and rhein were optimised using the CHARMM force field. Next, a docking protocol was performed to determine the interactions between the ligands and the proteins. This study was conducted using the free software AutoDock Vina, which calculates the mode of combination and affinity [24]. The scoring function was used to evaluate the binding intensity, with a smaller score representing stronger binding. Therefore, if the docking score was less than that of the experimental ligands, the corresponding potential target was selected for further studies.

### Network analysis for potential targets

The network construction is a key step in the network analysis. Before building the network, known targets of a given compound were collected from the STITCH (a database of known interactions between chemicals and proteins) [25]. Next, the known targets and the potential ones were integrated. They were mapped to several protein–protein interaction (PPI) databases, including BIOGRID, INTACT, MINT, DIP, BIND, and HPRD, by BisoGenet [26] to construct a target PPI network. Subsequently, an extended PPI (EPPI) network was further constructed by adding the nearest PPI neighbours. In these networks, each node is a protein, and two proteins are connected if there are interactions between them. The network visualisation was performed using Cytoscape (version 2.8) [27].

To reduce the false-positive rate in the molecular docking**,** a network analysis was then performed, and the topological parameters of the network were obtained. The network topological parameters, including the node degree, betweenness centrality, clustering coefficient, closeness centrality, and topological coefficient, reflect the structural relationship between each node in a network. These five topological parameters were calculated by the NetworkAnalyzer [28]. Next, the resulting receiver operating characteristic (ROC) curves of five topological parameters were plotted using GraphPad Prism (Version 6.01). The ROC curve, which could be used to evaluate the ability of topological parameters to identify targets, was a graphical plot with the false positive rate (FPR, i.e. 1-Specificity) as the horizontal axis and true positive rate (TPR, i.e. Sensitivity) as the vertical axis. Here, the FPR was the rate of potential targets considered as true targets, and the TPR was the rate of known targets considered as true targets. Subsequently, the network parameter with the largest area under the ROC curve (AUC) was selected to be the key parameter, and the best cut-off value of this parameter was determined to be the value with the largest Youden index (Youden index = Sensitivity + Specificity - 1). Finally, all of the potential targets with key parameter values greater than the cut-off value were selected.

### Enrichment analysis for potential targets

The enrichment analysis made it easy to associate proteins with biological processes. In this method, we assumed that potential target proteins would be selected as candidate targets if the enrichment analysis indicated that they were in the same biological process with known ones. Therefore, the enrichment analysis of the known and potential targets was performed using the DAVID tool [10]. The pathways with significant enrichment derived from the KEGG pathway database were selected if *p*-value < 0.05 [29]. Next, all potential targets in enriched pathways were eventually screened. These potential targets for final screening were defined as candidate targets, which meant that these targets were highly likely to be the true targets if experimentally proven.

### Experimental validation of the candidate targets

SPR is an important tool to determine the interactions between drugs and targets [30], and is widely used for detecting binding events, such as antibody–antigen, protein–protein, and receptor–ligand interactions [31, 32]. Binding experiments and kinetic analyses were performed using the PlexArray® HT system (Plexera®, LLC), based on SPR imaging (SPRi) at 25 °C with an injection rate of 2 μL·s^− 1^. The sample (object compound), positive control (rapamycin), and negative control (dimethyl sulphoxide) were printed on a 3D photo-crosslinking chip via a photo-crosslinking instrument (Amersham) [33]. The candidate protein solution in the running buffer (10 mM HEPES (pH 7.4), 150 mM NaCl, 0.005% Tween-20, and 3.4 mM EDTA) was used as the analyte at 375, 750, 1500, and 3000 nM by serial dilution. The sample injection cycle consisted of a 300 s association phase with an analyte solution and a 300 s dissociation phase with a running buffer. For the sensor chip regeneration, 10 mM glycine-HCl (pH 2.0, 3 μL·s^− 1^, 300 s) was then injected. All data were collected and monitored by the Plexera SPRi system and analysed using PlexeraDE software.

## Results

### Virtual screening based on ligand-protein docking

Ligand-protein docking was the first step in this study. Taking rhein as an example, 300 potential targets were quickly obtained from 2241 human protein targets by inverse molecular docking (Additional file 1: Table S1). However, many false positives could have existed in these 300 potential targets because of the low threshold present in the inverse docking. To decrease the false-positive rate, accurate molecular docking was used for further screening, reducing the number of potential targets to 67 (Additional file 1: Table S2).

### Virtual screening based on network analysis

**Fig. 2:**
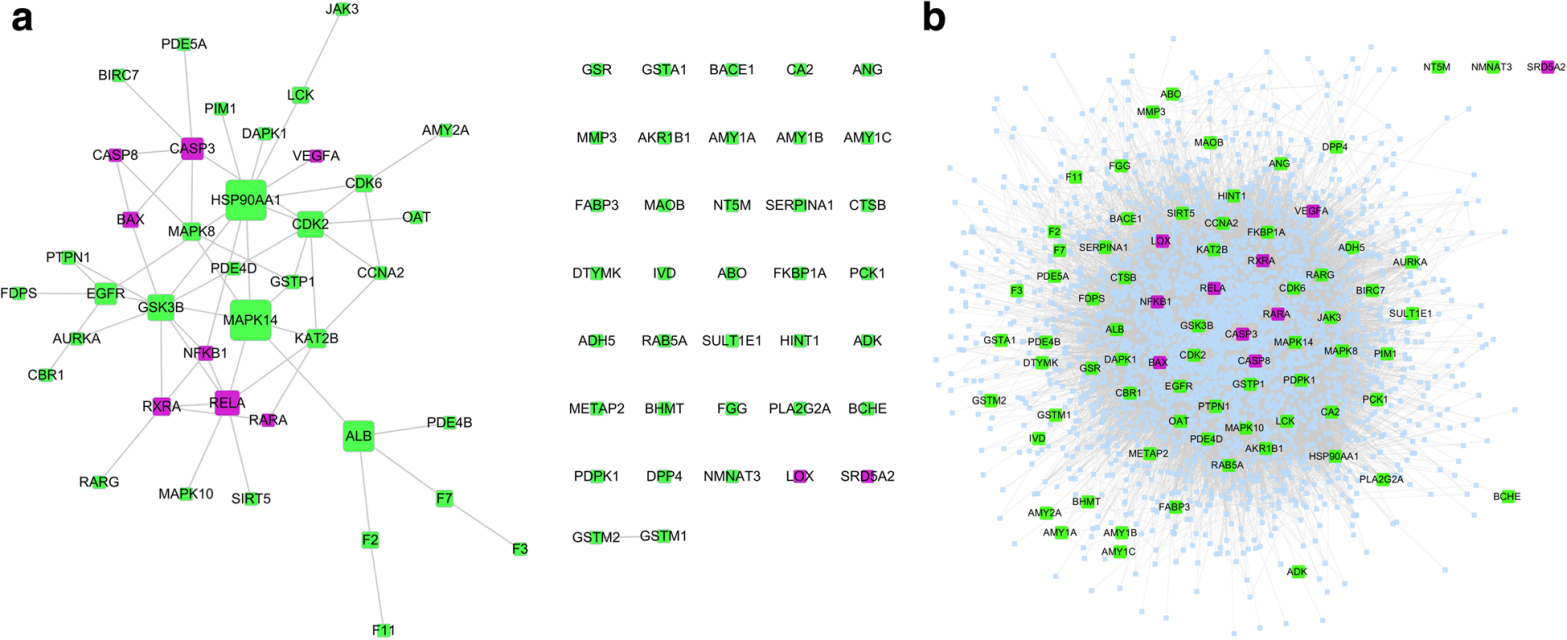
Network construction of rhein targets. **a** Rhein target protein–protein interaction network (PPI). **b** Extended rhein target PPI network (EPPI). In these networks, each node is a protein, and an edge indicates that two proteins interact with each other. Purple nodes represent known rhein targets; green nodes represent potential rhein targets; light blue nodes represent extended adjacent proteins of rhein targets

Network analysis was the second step. The PPI and EPPI networks was constructed after integrating potential and known targets of Rhein. Fig. 2a represents the integrated results of the 10 known targets (RXRA, CASP3, CASP8, BAX, LOX, RELA, NFKB1, VEGFA, RARA, and SRD5A2) and 67 potential targets. This network consisted of 77 nodes; more than half of the nodes were linked by 60 edges to form a cluster. As shown in Fig. 2b, the EPPI network included 3349 nodes and 66,348 edges; only three isolated nodes existed. Clearly, most of the known targets and potential targets had a close relationship with each other.

**Fig. 3:**
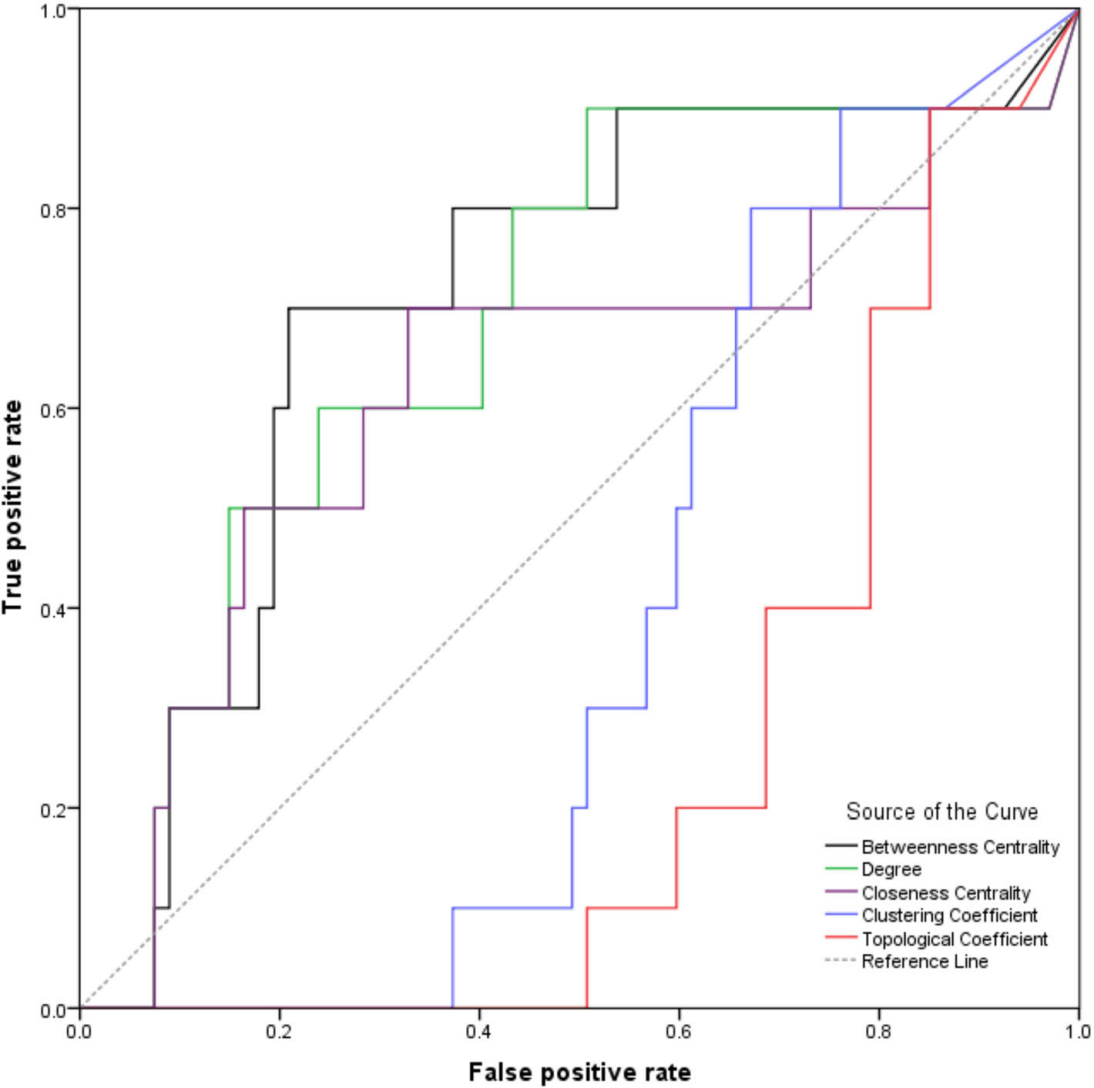
The receiver-operator characteristic (ROC) curves of five topological parameters in the extended protein–protein interaction (EPPI) network

In a complex network, the topology of the network carried a lot of important information that would help the target identification. Therefore, the degree, betweenness centrality, clustering coefficient, closeness centrality, and topological coefficient were chosen to further analyse the EPPI network to reduce the FPR. In the network analysis results (Additional file 1: Table S3), the ROC curves of betweenness centrality, degree, and closeness centrality were above the reference line, whereas the clustering coefficient and topological coefficient were under the reference line (Fig. 3). In this study, only the parameters above the reference line made sense. The betweenness centrality describes the capacity of carrying traffic; the degree reflects the importance of a node in the network; the closeness centrality represents the degree of closeness between a node and other nodes in the network [34]. The AUCs of all the network parameters were displayed in Table 1. Typically, the larger AUC value was corresponding to the better target identification ability for the parameter. Although all three parameters are critical, betweenness centrality was selected as the key parameter because it had the largest AUC (0.710). Finally, 21 nodes were screened because they were above the cut-off of betweenness centrality (0.0016) in the EPPI network. These 21 nodes included 7 known targets and 14 potential targets, and they were displayed in Table 2.

**Table 1:** Area under the ROC curve

Test Result Variable(s)	Area	Std. Error^a^	Asymptotic Sig.^b^	Asymptotic 95% Confidence Interval	
				Lower Bound	Upper Bound
Betweenness Centrality	.710	.090	.033	.533	.886
Degree	.690	.093	.054	.508	.871
Closeness Centrality	.627	.109	.198	.413	.841
Clustering Coefficient	.383	.068	.234	.250	.515
Topological Coefficient	.248	.059	.010	.133	.363

^a^Under the nonparametric assumption

^b^Null hypothesis: true area = 0.5

**Table 2:** 21 selected targets based on network analysis

Target Name	Gene Symbol	Target Type	Be Enriched or Not	Betweenness Centrality
Heat shock protein 90 kDa alpha (cytosolic), class A member 1	HSP90AA1	Candidate	Yes	0.04743
Epidermal growth factor receptor	EGFR	Candidate	Yes	0.02710
Cyclin-dependent kinase 2	CDK2	Candidate	Yes	0.01959
Albumin	ALB	Candidate	No	0.01653
Glycogen synthase kinase 3 beta	GSK3B	Candidate	Yes	0.01317
V-rel reticuloendotheliosis viral oncogene homolog A (avian)	RELA	Known	Yes	0.00777
Mitogen-activated protein kinase 14	MAPK14	Candidate	Yes	0.00765
Nuclear factor of kappa light polypeptide gene enhancer in B-cells 1	NFKB1	Known	Yes	0.00364
Dipeptidyl-peptidase 4	DPP4	Candidate	No	0.00348
Mitogen-activated protein kinase 8	MAPK8	Candidate	Yes	0.00321
Lymphocyte-specific protein tyrosine kinase	LCK	Candidate	Yes	0.00279
Cyclin-dependent kinase 6	CDK6	Candidate	Yes	0.00277
RAB5A, member RAS oncogene family	RAB5A	Candidate	No	0.00275
Serpin peptidase inhibitor, clade A (alpha-1 antiproteinase, antitrypsin), member 1	SERPINA1	Candidate	No	0.00244
Cathepsin B	CTSB	Candidate	No	0.00241
Caspase 3, apoptosis-related cysteine peptidase	CASP3	Known	Yes	0.00238
K(lysine) acetyltransferase 2B	KAT2B	Candidate	No	0.00237
Retinoic acid receptor, alpha	RARA	Known	Yes	0.00198
Vascular endothelial growth factor A	VEGFA	Known	Yes	0.00178
Caspase 8, apoptosis-related cysteine peptidase	CASP8	Known	Yes	0.00165
Retinoid X receptor, alpha	RXRA	Known	Yes	0.00163

### Virtual screening based on enrichment analysis

Enrichment analysis was the third step to supplement the deficiency of network analysis for identifying targets. As a result, 15 out of 21 proteins were enriched including 6 known targets (RELA, NFKB1, CASP3, CASP8, RXRA, and VEGFA) and 9 potential ones (LCK, HSP90AA1, RAB5A, EGFR, CDK2, CDK6, GSK3B, MAPK8, and MAPK14). Thus, these 9 potential targets were regarded as rhein candidate targets. In addition, all 15 proteins were respectively present in 11 items in KEGG pathways (see Additional file 1: Table S4).

### SPR experimental validation for rhein candidate targets

**Fig. 4:**
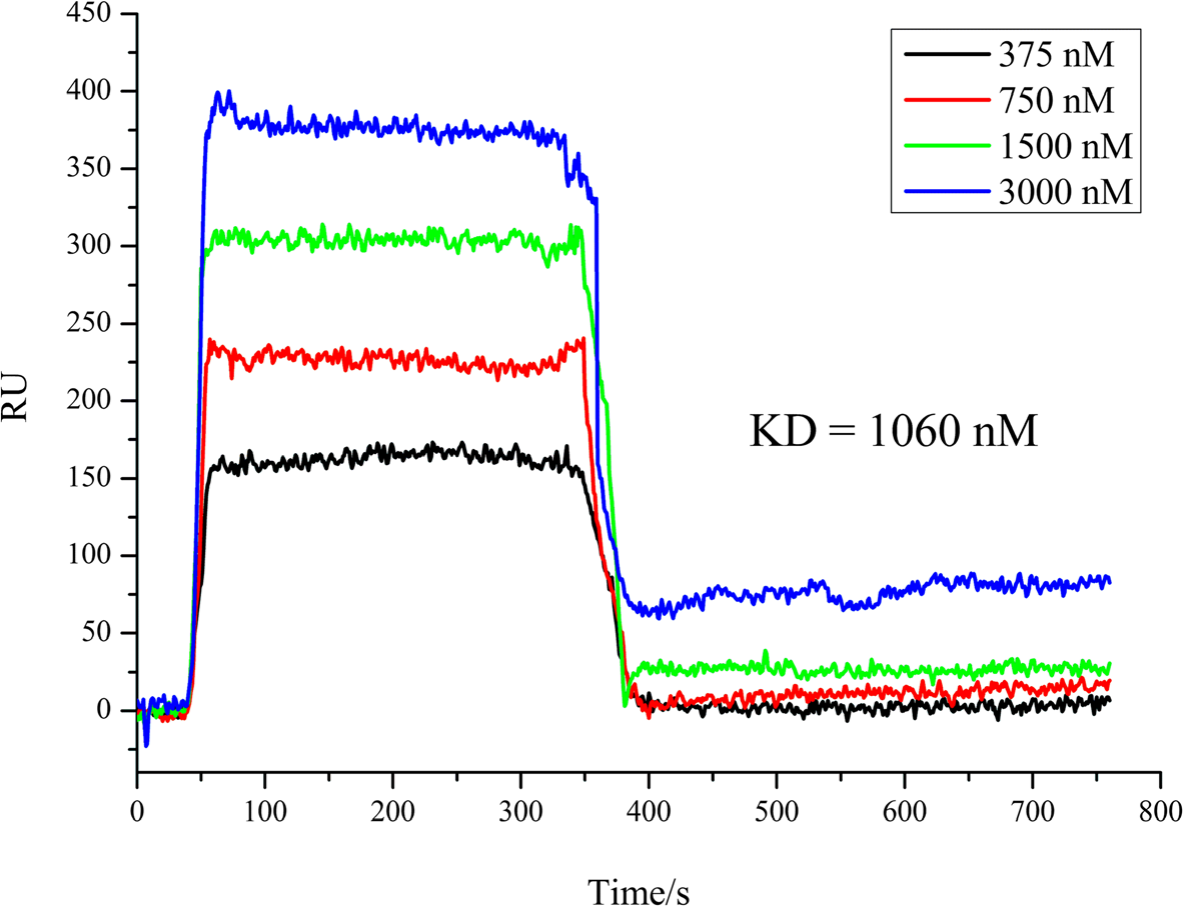
The surface plasmon resonance (SPR) results of the interaction between LCK and rhein. Increased concentration of LCK protein showed a trend of increased binding with rhein; the equilibrium dissociation constant (*K*_*D*_) was 1.060 × 10^− 6^ M

According to the literature search results, 5 of the 9 candidate targets, including EGFR [35, 36], MAPK8 [17], MAPK14 [37], CDK6 [38], and HSP90AA1 [15], had been previously reported, in spite of not being included in the STITCH database. Therefore, the remaining four candidate targets (LCK, RAB5A, CDK2, and GSK3B) were selected for further research using SPR. The positive and negative control signals were shown in supplementary materials (Additional file 1: Figure S1), which indicated that the sensor chip quality was normal. In the experimental results, for LCK, the binding tendency to rhein increased with increasing the concentration of the protein, whereas for RAB5A, CDK2, and GSK3B, the tendency was not obvious. The binding curves of rhein with LCK were shown in Fig. 4. The kinetic parameters were fitted and obtained using the LCK signals bound with rhein. The association rate constant (*k*_*a*_), dissociation rate constant (*k*_*d*_), and equilibrium dissociation constant (*K*_*D*_) were 186 (M·s)^− 1^, 1.97 × 10^− 4^ s^− 1^, and 1.060 × 10^− 6^ M, respectively. Therefore, after the experimental verification of SPR, we had reason to believe that LCK was a new target of rhein.

## Discussion

At present, there had been many successful cases of ligand-protein docking for target identification [39, 40]. The use of ligand-protein docking provided the conditions for the rapid screening of potential targets, rather than the aimless trial of luck. In this study, virtual screening based on ligand-protein docking was divided into two steps. The first step was inverse rhein molecular docking analysis. In this step, 300 potential targets were selected from 2241 human protein targets. The second step was the accurate rhein molecular docking analysis. In this step, the 300 targets were further reduced to 67 potential targets. These two steps were designed to reduce the rate of false positives and obtain more accurate targets. Although the ligand-protein docking was popular for drug target identification, challenges remained for this method due to its limitations that included insufficiencies of the database resources, imperfections of the scoring functions, and inaccurate selection of binding sites and docking poses [41]. Due to these limitations, there may still be a few false positives among the 67 potential targets. In addition, the direct verification of 67 potential targets by experiments was time-consuming and costly. Therefore, a further method was needed to screen potential targets and reduce false positive targets.

**Fig. 5:**
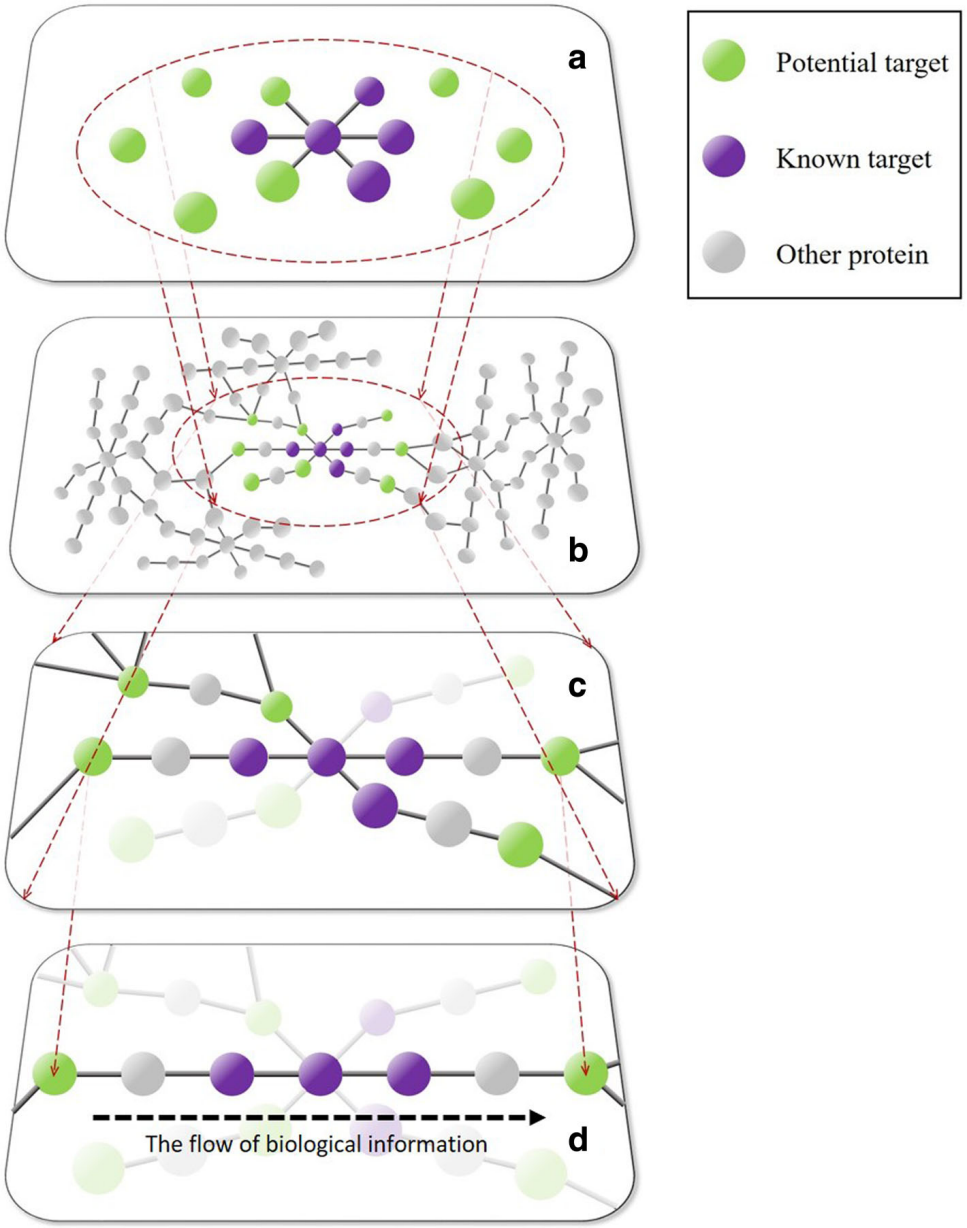
Diagrammatic sketch of the idea for network analysis and enrichment analysis. In this diagrammatic sketch, plane **a** represents the target protein–protein interaction (PPI) of one bioactive compound, targets of which were mapped to a biological network (plane **b**). In fact, the target extended PPI (EPPI) of this bioactive compound is the network with broken circle in plane **b**. According to the importance of nodes in the network, plane **c** was selected from the EPPI via network analysis. The plane **d** represents the enriched pathway of proteins in plane **c**. Thus, the potential targets of this bioactive compound in plane **d** could be considered to be candidate targets

The network analysis was a new strategy to comprehensively screen drug targets [8]. In biological networks, the targets of one bioactive compound always gathered in a cluster. For instance, there were close interactions between the targets of nearly any bioactive compound in the STITCH database [42], which meant that the adjacent nodes of a known target were likely to be a target as well. To clearly illustrate the principle of network analysis, the diagrammatic sketch of the idea was constructed as shown in Fig. 5. In this diagrammatic sketch, plane a represented the target PPI of one bioactive compound, targets of which were mapped to a biological network (plane b). All the known targets of this bioactive compound clustered together, and the target EPPI of this compound was the network with broken circle in plane b. Then, the plane c was selected from the EPPI according to the importance of nodes in the EPPI network. Thus, the potential targets in plane c were used for further screen. In this study, the PPI network of rhein targets was consisted of a big cluster with 40 nodes linked by 60 interactions along with 37 isolated nodes. Further research should consider whether these 37 isolated nodes were connected to other known targets via neighbouring nodes such that one whole cluster forms. Certainly, each node in the cluster had a high probability of being a target. Therefore, the EPPI network was further constructed to filter targets. Topological characteristics offered significant insight into biologically relevant connectivity patterns, and pinpoint likely key targets in the network [43]. The node degree represented the number of other nodes connected to a node. A high degree node was generally considered to be important because of its extensive connectivity [44, 45]. Similarly, the closeness centrality represented the degree of closeness between a node and other nodes in the network. The node with a large closeness centrality was also a protein of great importance. The betweenness centrality was another basic property of a network. The node with a large betweenness centrality was always a key transmit point for biological information flow; if this node was lost or blocked in a network, it resulted in the emergence of many modules [34, 46]. Here, betweenness centrality was determined as a key parameter because it had the largest AUC (0.710), which implied the best predictive rate. Then, the 21 nodes were screened according to the highest cut-off (0.0016) of betweenness centrality. These 21 nodes included 7 known targets and 14 potential targets. Examples used in this study demonstrated that our network analysis method was very efficient, reducing 67 potential targets to 14 ones. However, our network analysis needed two prerequisite conditions: 1. There must be a certain number of known targets; 2. There should be direct or indirect links between known and potential targets. In other words, if the number of known targets was insufficient enough or the known targets were not closely related to potential targets, the false positives or false negatives might increase in the results. In addition, the network analysis could not reflect the flow of biological information because the network used in network analysis was usually undirected. Therefore, the enrichment analysis was another required method in order to further reduce the false positives and to consider the flow of bio-information closer to reality.

**Fig. 6:**
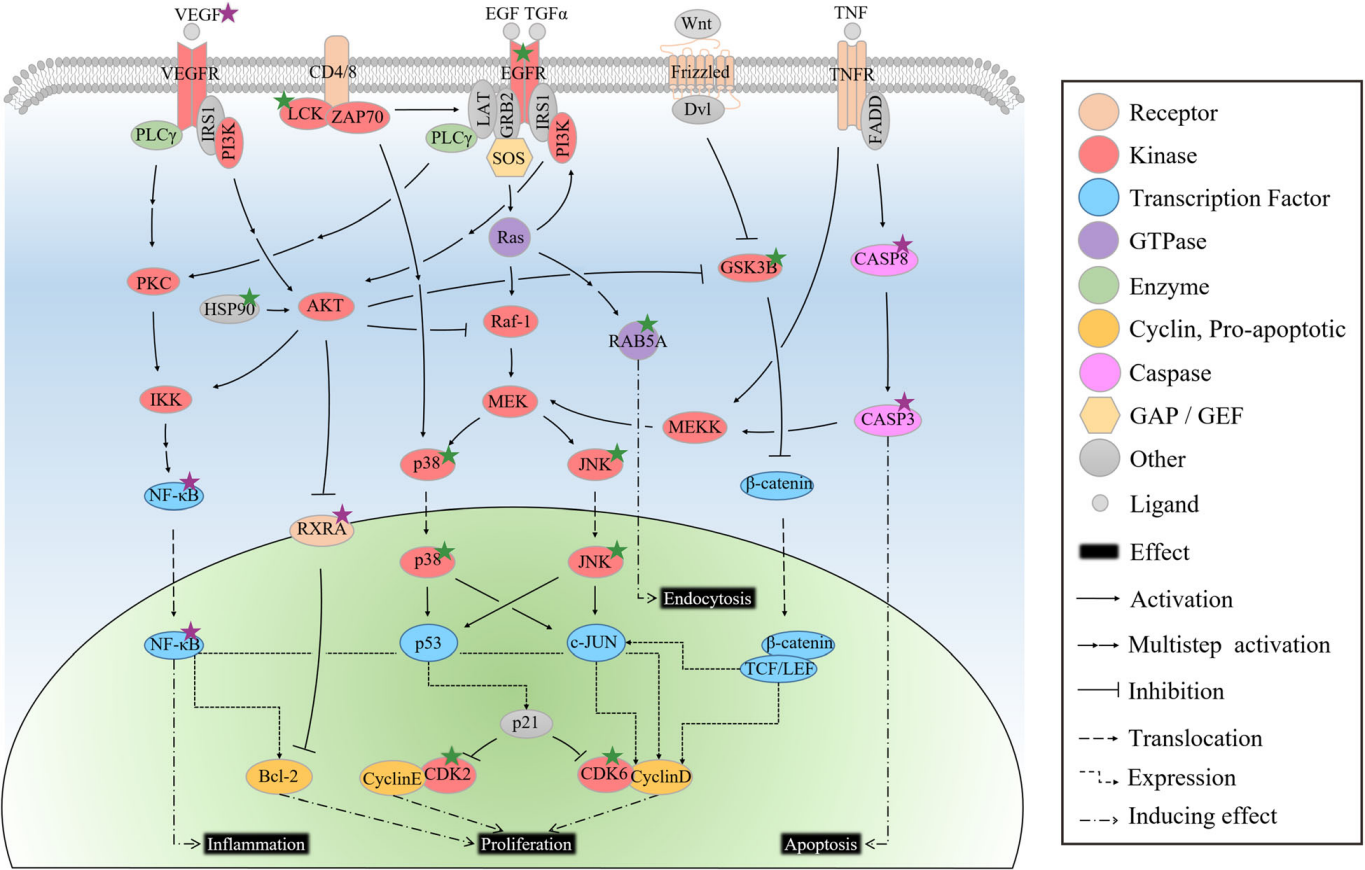
The integrated network of enrichment pathways of rhein targets. This pathway was constructed via manually extracting the biological process which is related to enriched targets of rhein from the KEGG pathway. The main body of a biological process was extracted if a rhein target was in this biological process. The protein marked by star is the rhein target. Purple and green stars represent known and candidate targets, respectively

The pathway enrichment analysis was usually used to assess the distribution of given proteins in the KEGG pathway and determine their contribution to biological processes. This method would calculate the hypergeometric distributions between given proteins and pathways and return a *P*-value for each pathway in which the given proteins existed. Based on the *P*-value, it was assessed whether the given proteins were enriched in that pathway [10]. Obviously, the enrichment analysis had significant implications for establishing the relationships between proteins and pathways. Here, the enrichment analysis was innovatively used for the target identification since the enriched proteins often played similar and important biological roles in the biological process, and were likely to be the targets of the bioactive molecule. For example, the activation of JAK2 and STAT3 induced the expression of TNF-α and IL-6 in acute renal injury, while curcumin protected against the acute renal injury by distinctly inhibiting the activation of JAK2 and STAT3 in the JAK2/STAT3 pathway [47]. As shown in Fig. 5, the proteins with the flow of biological information in plane d were enriched from plane c, and thus the range of potential targets would be more accurate after the enrichment analysis. In this study, 21 proteins from the network analysis screening were subjected to the enrichment analysis. The results showed that 15 proteins were enriched and 9 of the 15 proteins were potential targets and determined to be candidate targets. Interestingly, 5 of the 9 candidate targets had been previously reported, in spite of not being included in the STITCH database. This situation further verified the accuracy and reliability of the integration strategy used in this study. Moreover, 11 KEGG pathways that were significantly enriched interacted closely through the 15 enriched proteins, as shown in Fig. 6. All 11 KEGG pathways were associated with inflammation, proliferation and apoptosis, which were consistent with the pharmacological activities of rhein, again suggesting that each enriched protein was likely to be a target.

LCK, a member of the Src family of protein tyrosine kinases [48], was a new rhein target identified by our strategy. Our SPR experiment revealed that LCK could interact with rhein, and the binding tendency was proportional to the protein concentration. In biological systems, LCK played an important role in the T-cell antigen receptor (TCR)-linked signal transduction pathway as a non-receptor tyrosine kinase [49]. LCK constitutively associated with the cytoplasmic portions of the CD4 and CD8 surface receptors, and then initiated the TCR-linked signaling pathway [50]. Upon TCR stimulation, LCK phosphorylated the TCR, thus leading to the recruitment, phosphorylation, and activation of ZAP70 [51]. Activated ZAP70 then directly or indirectly regulated the MAPK and the NFKB signalling pathways, subsequently affecting cell proliferation and inflammatory processes [52, 53]. As a new target of rhein, LCK might play an important role in the treatment of cancer or inflammation. Of course, the therapeutic effect of rhein was not only due to regulating the LCK target, but also was the result of synergistic and comprehensive regulation of multiple targets in different pathways [13]. Rhein could inhibit the phosphorylation of EGFR, p38 and JNK in the classical MAPK cascade [17, 35–37], repress the activity of RELA and NFKB1 in the NF-κB signalling pathway [17, 54–56], promote apoptosis through the activation of CASP3 and CASP8 in the apoptotic pathway [57], induce G0/G1 arrest through CDK6 inhibition in the cell cycle [38], decrease the expression of VEGFA and the activity of HSP90AA1 and RXRA in other pathways [14, 15, 58]. Apparently, the rhein-mediated biological network was vast and complex. The therapeutic effect of rhein was the synergistic and comprehensive result of this vast and complex network [13], and the perturbation of multiple targets gave rhein a variable and effective pharmacological activity.

## Conclusion

In this study, ligand-protein docking, network analysis, and enrichment analysis were integrated to identify new targets of rhein, followed by the validation of these targets using SPR experiments. Although any one of these methods had been applied to the target identification before, the rational combination of them for the target identification was novel. The integrated network of enriched pathways was used to elucidate the comprehensive pharmacological mechanisms of rhein. This study provided a new strategy to effectively identify candidate targets and infer the molecular mechanisms of bioactive compounds.

## Additional file

Additional file 1: **Table S1**. Inverse Docking Result. **Table S2**. Potential Targets of Rhein after Accurate Molecular Docking. **Table S3**. Sorting results of topological parameters. **Table S4.** 15 Enriched Proteins in 11 KEGG Pathways. **Figure S1**. The positive and negative control signal for SPR. (PDF 637 kb)

## Abbreviations

AUC: Area under the curve
EPPI: Extended PPI
FPR: False positive rate
PPI: Protein–protein interaction
ROC: Receiver operating characteristic
SPR: Surface plasmon resonance
TPR: True positive rate
