# Supplementary material for: An integrated strategy for identifying new targets and inferring the mechanism of action: taking rhein as an example
Source: BMC Bioinformatics. 2018 Sep 6;19:315. doi: 10.1186/s12859-018-2346-4 (PMC6127921; doi:10.1186/s12859-018-2346-4)

An Integrated Strategy for Identifying New Targets and Inferring the Mechanism of Action: Taking Rhein as an Example

Hao Sun[1,2], Yiting Shen[1], Guangwen Luo[1], Yuepiao Cai[1*], Zheng Xiang[1*]

[1]School of Pharmaceutical Sciences, Wenzhou Medical University, Wenzhou 325035, China

[2]Pharmacy Department, Women's Hospital, Zhejiang University School of Medicine, Hangzhou 310006, Zhejiang, China

Author e-mails:

Hao Sun, SH1813@163.com

Yiting Shen, 594789144@qq.com

Guangwen Luo, 841907612@qq.com

*Corresponding author:

Zheng Xiang

Tel/Fax: 86-577-86689156

E-mail address: xzh0077@126.com

Yuepiao Cai

Tel/Fax: 86-577-86689156

E-mail address: ypcai@wmu.edu.cn

Table S1. Inverse Docking Result

Pharma Model	Name	Uniplot	Fit
3c4f	Basic fibroblast growth factor receptor 1	FGFR1_HUMAN	3.945
1q5h	Deoxyuridine 5-triphosphate nucleotidohydrolase, mitochondrial	DUT_HUMAN	3.82
1r1h	Neprilysin	NEP_HUMAN	3.753
1tdi	Glutathione S-transferase A3	Q16772	3.714
1boa	Methionine aminopeptidase 2	AMPM2_HUMAN	3.703
1udw	Uridine-cytidine kinase 2	Q9BZX2	3.661
1jtv	Estradiol 17-beta-dehydrogenase 1	P14061	3.65
1kav	Tyrosine-protein phosphatase non-receptor type 1	PTN1_HUMAN	3.643
1i9p	Carbonic anhydrase 2	CAH2_HUMAN	3.623
1nhz	Glucocorticoid receptor	GCR_HUMAN	3.613
1kpf	Histidine triad nucleotide-binding protein 1	HINT1_HUMAN	3.612
1cm0	Histone acetyltransferase PCAF	Q92831	3.61
1gs4	Androgen receptor	ANDR_HUMAN	3.601
1qcf	Tyrosine-protein kinase HCK	HCK_HUMAN	3.592
2oah	Beta-secretase 1	BACE1_HUMAN	3.576
1hfv	NONE	NONE	3.575
1q1z	Sulfotransferase family cytosolic 2B member 1	O00204	3.557
1b59	Methionine aminopeptidase 2	AMPM2_HUMAN	3.547
1b55	Tyrosine-protein kinase BTK	BTK_HUMAN	3.545
1oiq	Cell division protein kinase 2	P24941	3.532
1g53	Carbonic anhydrase 2	CAH2_HUMAN	3.527
1uyg	Heat shock protein HSP 90-alpha	HS90A_HUMAN	3.524
1zzl	Mitogen-activated protein kinase 14	Q16539	3.522
1osf	Heat shock protein HSP 90-alpha	HS90A_HUMAN	3.521
1guh	Glutathione S-transferase A1	P08263	3.515
1bnv	Carbonic anhydrase 2	CAH2_HUMAN	3.514
1d2s	Sex hormone-binding globulin	SHBG_HUMAN	3.512
1jf7	Tyrosine-protein phosphatase non-receptor type 1	PTN1_HUMAN	3.508
1xon	cAMP-specific 3,5-cyclic phosphodiesterase 4D	PDE4D_HUMAN	3.506
1jk7	Serine/threonine-protein phosphatase PP1-gamma catalytic subunit	P36873	3.5
1o4h	Proto-oncogene tyrosine-protein kinase Src	SRC_HUMAN	3.491
1dug	Fibrinogen gamma chain	FIBG_HUMAN	3.474
1qpe	Proto-oncogene tyrosine-protein kinase LCK	LCK_HUMAN	3.469
1nuh	Glucose-6-phosphate isomerase	G6PI_HUMAN	3.468
2is0	Beta-secretase 1	BACE1_HUMAN	3.459
1m51	Phosphoenolpyruvate carboxykinase, cytosolic [GTP]	P35558	3.458
1bl4	Peptidyl-prolyl cis-trans isomerase FKBP1A	FKB1A_HUMAN	3.44
1hrk	Ferrochelatase, mitochondrial	P22830	3.427
1xdc	Superoxide dismutase [Mn], mitochondrial	P04179	3.42
2dux	Aldose reductase	ALDR_HUMAN	3.416
1e9d	Thymidylate kinase	KTHY_HUMAN	3.404
2p8h	Beta-secretase 1	BACE1_HUMAN	3.383

1bmn	Prothrombin	THRB_HUMAN	3.382
1fkn	Beta-secretase 1	BACE1_HUMAN	3.38
1o4r	Proto-oncogene tyrosine-protein kinase Src	SRC_HUMAN	3.379
1j4i	Peptidyl-prolyl cis-trans isomerase FKBP1A	FKB1A_HUMAN	3.362
1uy6	Heat shock protein HSP 90-alpha	HS90A_HUMAN	3.305
1yc1	Heat shock protein HSP 90-alpha	HS90A_HUMAN	3.302
2c6i	Cell division protein kinase 2	P24941	3.299
1j4h	Peptidyl-prolyl cis-trans isomerase FKBP1A	FKB1A_HUMAN	3.298
2irz	Beta-secretase 1	BACE1_HUMAN	3.295
1jkl	Death-associated protein kinase 1	DAPK1_HUMAN	3.291
3f4x	Carbonic anhydrase 2	P00918	3.29
1v1k	Cell division protein kinase 2	P24941	3.289
1yz3	Phenylethanolamine N-methyltransferase	PNMT_HUMAN	3.288
2zi2	Prothrombin	P00734	3.288
1tqf	Beta-secretase 1	BACE1_HUMAN	3.281
1r1i	Neprilysin	NEP_HUMAN	3.277
1nn0	Thymidylate kinase	KTHY_HUMAN	3.27
1yj6	Glutathione S-transferase Mu 1	GSTM1_HUMAN	3.263
2fpy	Dihydroorotate dehydrogenase, mitochondrial	PYRD_HUMAN	3.253
1xo2	Cell division protein kinase 6	CDK6_HUMAN	3.241
3fv8	Mitogen-activated protein kinase 10	P53779	3.225
3f82	Hepatocyte growth factor receptor	P08581	3.219
1fki	Peptidyl-prolyl cis-trans isomerase FKBP1A	FKB1A_HUMAN	3.217
3duy	Beta-secretase 1	P56817	3.209
1dnc	Glutathione reductase, mitochondrial	P00390	3.206
1zpb	Coagulation factor XI	FA11_HUMAN	3.197
2ph6	Beta-secretase 1	BACE1_HUMAN	3.182
1u32	Serine/threonine-protein phosphatase PP1-gamma catalytic subunit	P36873	3.179
1q20	Sulfotransferase family cytosolic 2B member 1	O00204	3.175
1bn3	Carbonic anhydrase 2	CAH2_HUMAN	3.173
1hms	Fatty acid-binding protein, heart	FABPH_HUMAN	3.168
1gnj	Serum albumin	ALBU_HUMAN	3.168
2can	Ornithine aminotransferase, mitochondrial	OAT_HUMAN	3.163
1v4s	Glucokinase	HXK4_HUMAN	3.158
1gsf	Glutathione S-transferase A1	P08263	3.152
1b1i	Angiogenin	ANGI_HUMAN	3.151
1y2k	cAMP-specific 3,5-cyclic phosphodiesterase 4D	PDE4D_HUMAN	3.15
1gw6	Leukotriene A-4 hydrolase	LKHA4_HUMAN	3.145
1rlb	Transthyretin	TTHY_HUMAN	3.13
1z6t	Apoptotic protease-activating factor 1	APAF_HUMAN	3.112
1di8	Cell division protein kinase 2	P24941	3.112
1gre	Glutathione reductase, mitochondrial	P00390	3.11
1kta	Branched-chain-amino-acid aminotransferase, mitochondrial	O15382	3.109
1ls6	Sulfotransferase 1A1	ST1A1_HUMAN	3.1

1og5	Cytochrome P450 2C9	CP2C9_HUMAN	3.09
2pe1	3-phosphoinositide-dependent protein kinase 1	PDPK1_HUMAN	3.088
1hne	Leukocyte elastase	ELNE_HUMAN	3.081
1db1	Vitamin D3 receptor	VDR_HUMAN	3.077
1t32	Cathepsin G	CATG_HUMAN	3.077
1y2e	cAMP-specific 3,5-cyclic phosphodiesterase 4D	PDE4D_HUMAN	3.075
2auh	Insulin receptor	INSR_HUMAN	3.057
1ciz	Stromelysin-1	MMP3_HUMAN	3.052
2c6t	Cyclin-A2	CCNA2_HUMAN	3.046
1pso	NONE	NONE	3.04
1no6	Tyrosine-protein phosphatase non-receptor type 1	PTN1_HUMAN	3.036
1o43	Proto-oncogene tyrosine-protein kinase Src	SRC_HUMAN	3.035
1bnw	Carbonic anhydrase 2	CAH2_HUMAN	3.032
1i9o	Carbonic anhydrase 2	CAH2_HUMAN	3.024
1i9n	Carbonic anhydrase 2	CAH2_HUMAN	3.004
3dv1	Beta-secretase 1	P56817	2.997
2of0	Beta-secretase 1	BACE1_HUMAN	2.996
1s9j	Dual specificity mitogen-activated protein kinase kinase 1	MP2K1_HUMAN	2.995
1h9u	Retinoic acid receptor RXR-beta	RXRB_HUMAN	2.986
1tu6	Cathepsin K	CATK_HUMAN	2.986
1t46	Mast/stem cell growth factor receptor	KIT_HUMAN	2.986
1ywn	Vascular endothelial growth factor receptor 2	VGFR2_HUMAN	2.985
1fcx	Retinoic acid receptor gamma	RARG_HUMAN	2.985
17gs	Glutathione S-transferase P	GSTP1_HUMAN	2.984
1hs6	Leukotriene A-4 hydrolase	LKHA4_HUMAN	2.983
1z6e	Coagulation factor X	FA10_HUMAN	2.982
2i3h	Baculoviral IAP repeat-containing protein 7	Q96CA5	2.982
1fd0	Retinoic acid receptor gamma	RARG_HUMAN	2.979
1nus	Nicotinamide mononucleotide adenylyltransferase 3	Q96T66	2.978
2q2z	Kinesin-like protein KIF11	KIF11_HUMAN	2.978
1w8l	Peptidyl-prolyl cis-trans isomerase A	P62937	2.978
2fl2	Kinesin-like protein KIF11	KIF11_HUMAN	2.977
3e92	Mitogen-activated protein kinase 14	Q16539	2.977
1kao	Ras-related protein Rap-2a	P10114	2.977
2fky	Kinesin-like protein KIF11	KIF11_HUMAN	2.975
1xh0	Pancreatic alpha-amylase	AMYP_HUMAN	2.974
2oh4	Vascular endothelial growth factor receptor 2	P35968	2.974
1ro9	cAMP-specific 3,5-cyclic phosphodiesterase 4B	PDE4B_HUMAN	2.974
2p1u	Retinoic acid receptor RXR-alpha	RXRA_HUMAN	2.973
1yw7	Methionine aminopeptidase 2	AMPM2_HUMAN	2.972
2iw6	Cyclin-A2	CCNA2_HUMAN	2.972
2g1q	Kinesin-like protein KIF11	KIF11_HUMAN	2.971
1if6	Carbonic anhydrase 2	CAH2_HUMAN	2.971
1xap	Retinoic acid receptor beta	RARB_HUMAN	2.97

1e9a	Thymidylate kinase	KTHY_HUMAN	2.97
1g3m	Estrogen sulfotransferase	ST1E1_HUMAN	2.969
1exa	Retinoic acid receptor gamma	RARG_HUMAN	2.968
2bk3	Amine oxidase [flavin-containing] B	AOFB_HUMAN	2.966
1md3	Glutathione S-transferase P	GSTP1_HUMAN	2.966
1uki	Mitogen-activated protein kinase 8	MK08_HUMAN	2.966
1lhv	Sex hormone-binding globulin	SHBG_HUMAN	2.966
3f7z	Glycogen synthase kinase-3 beta	P49841	2.965
1pic	Phosphatidylinositol 3-kinase regulatory subunit alpha	P85A_HUMAN	2.964
1h07	Cell division protein kinase 2	P24941	2.962
2oqv	Dipeptidyl peptidase 4	DPP4_HUMAN	2.962
3bm9	Heat shock protein HSP 90-alpha	HS90A_HUMAN	2.961
1x98	Aldose reductase	ALDR_HUMAN	2.961
3c4c	B-Raf proto-oncogene serine/threonine-protein kinase	BRAF1_HUMAN	2.96
1skx	Nuclear receptor subfamily 1 group I member 2	NR1I2_HUMAN	2.96
1md4	Glutathione S-transferase P	GSTP1_HUMAN	2.96
3dtw	Vascular endothelial growth factor receptor 2	P35968	2.959
1m2z	Glucocorticoid receptor	GCR_HUMAN	2.959
1h01	Cell division protein kinase 2	P24941	2.958
1tfg	Transforming growth factor beta-2	TGFB2_HUMAN	2.958
2pe2	3-phosphoinositide-dependent protein kinase 1	PDPK1_HUMAN	2.957
1q4x	Thyroid hormone receptor beta	P10828	2.957
3b5r	Androgen receptor	ANDR_HUMAN	2.956
1rwq	Dipeptidyl peptidase 4	DPP4_HUMAN	2.956
1oir	Cell division protein kinase 2	P24941	2.955
1ju6	Thymidylate synthase	TYSY_HUMAN	2.954
2no3	Mitogen-activated protein kinase 8	MK08_HUMAN	2.954
2v12	Renin	RENI_HUMAN	2.952
1lzj	B transferase	Q9NY01	2.952
1i5r	Estradiol 17-beta-dehydrogenase 1	P14061	2.951
2wi5	NONE	NONE	2.951
1ph0	Tyrosine-protein phosphatase non-receptor type 1	PTN1_HUMAN	2.951
1di9	Mitogen-activated protein kinase 14	Q16539	2.95
1bnq	Carbonic anhydrase 2	CAH2_HUMAN	2.95
2rg5	Mitogen-activated protein kinase 14	Q16539	2.95
1m6d	Cathepsin F	Q9UBX1	2.949
1q91	5(3)-deoxyribonucleotidase, mitochondrial	Q9NPB1	2.948
1nmy	Thymidylate kinase	KTHY_HUMAN	2.948
1wt1	Histo-blood group ABO system transferase	P16442	2.948
1dic	Complement factor D	CFAD_HUMAN	2.947
2ito	Epidermal growth factor receptor	EGFR_HUMAN	2.947
1ice	Caspase-1	CASP1_HUMAN	2.947
3fc1	Mitogen-activated protein kinase 14	Q16539	2.946
1fby	Retinoic acid receptor RXR-alpha	RXRA_HUMAN	2.946

1wma	Carbonyl reductase [NADPH] 1	CBR1_HUMAN	2.946
2hyy	Proto-oncogene tyrosine-protein kinase ABL1	P00519	2.945
3f7i	Baculoviral IAP repeat-containing protein 7	Q96CA5	2.945
1q4n	Alpha-amylase 1	P04745	2.944
3dzy	Retinoic acid receptor RXR-alpha	P19793	2.944
1ua2	Cell division protein kinase 7	P50613	2.944
1d5j	Stromelysin-1	MMP3_HUMAN	2.943
1s19	Vitamin D3 receptor	VDR_HUMAN	2.942
1nwe	Tyrosine-protein phosphatase non-receptor type 1	PTN1_HUMAN	2.942
1q3d	Glycogen synthase kinase-3 beta	GSK3B_HUMAN	2.941
1biw	Stromelysin-1	MMP3_HUMAN	2.941
1o41	Proto-oncogene tyrosine-protein kinase Src	SRC_HUMAN	2.941
2bkz	Cyclin-A2	CCNA2_HUMAN	2.941
2pgt	Glutathione S-transferase P	GSTP1_HUMAN	2.941
1b6a	Methionine aminopeptidase 2	AMPM2_HUMAN	2.941
1qpf	Peptidyl-prolyl cis-trans isomerase FKBP1A	FKB1A_HUMAN	2.94
1lzi	Histo-blood group ABO system transferase	P16442	2.94
1xha	cAMP-dependent protein kinase catalytic subunit alpha	P00517	2.938
1e3g	Androgen receptor	ANDR_HUMAN	2.938
1ydt	cAMP-dependent protein kinase catalytic subunit alpha	P00517	2.938
1r6u	Tryptophanyl-tRNA synthetase, cytoplasmic	P23381	2.938
1m6w	Alcohol dehydrogenase class-3	ADHX_HUMAN	2.937
1n1m	Dipeptidyl peptidase 4	DPP4_HUMAN	2.936
1y8y	Cell division protein kinase 2	P24941	2.936
1lt8	Betaine--homocysteine S-methyltransferase 1	Q93088	2.935
1p2s	GTPase HRas	RASH_HUMAN	2.934
1dan	Coagulation factor VII	FA7_HUMAN	2.934
1itu	Dipeptidase 1	DPEP1_HUMAN	2.933
1gmy	Cathepsin B	CATB_HUMAN	2.932
1y6b	Vascular endothelial growth factor receptor 2	VGFR2_HUMAN	2.932
2aa6	Mineralocorticoid receptor	MCR_HUMAN	2.932
1gsn	Glutathione reductase, mitochondrial	P00390	2.932
1r0p	Hepatocyte growth factor receptor	MET_HUMAN	2.932
9gss	Glutathione S-transferase P	GSTP1_HUMAN	2.931
1r81	Histo-blood group ABO system transferase	P16442	2.931
2gfs	Mitogen-activated protein kinase 14	Q16539	2.931
1jqd	Histamine N-methyltransferase	P50135	2.931
3ekr	Heat shock protein HSP 90-alpha	P07900	2.93
2o9i	Nuclear receptor subfamily 1 group I member 2	NR1I2_HUMAN	2.93
2f57	Serine/threonine-protein kinase PAK 7	PAK7_HUMAN	2.93
1fvv	Cyclin-A2	CCNA2_HUMAN	2.929
1o4p	Proto-oncogene tyrosine-protein kinase Src	SRC_HUMAN	2.929
3bbt	Receptor tyrosine-protein kinase erbB-4	ERBB4_HUMAN	2.929
2pou	Carbonic anhydrase 2	CAH2_HUMAN	2.928

1lhn	Sex hormone-binding globulin	SHBG_HUMAN	2.928
2zb1	Mitogen-activated protein kinase 14	Q16539	2.927
1hmp	Hypoxanthine-guanine phosphoribosyltransferase	P00492	2.926
1r7u	Histo-blood group ABO system transferase	P16442	2.925
1fkf	Peptidyl-prolyl cis-trans isomerase FKBP1A	FKB1A_HUMAN	2.924
3b4f	Carbonic anhydrase 2	CAH2_HUMAN	2.924
1qpl	Peptidyl-prolyl cis-trans isomerase FKBP1A	FKB1A_HUMAN	2.923
1kv1	Mitogen-activated protein kinase 14	Q16539	2.922
1e5a	Transthyretin	TTHY_HUMAN	2.922
1pmv	Mitogen-activated protein kinase 10	MK10_HUMAN	2.922
1jj7	Antigen peptide transporter 1	Q03518	2.921
1kqu	Phospholipase A2, membrane associated	P14555	2.921
1csb	Cathepsin B	CATB_HUMAN	2.921
2hoc	Carbonic anhydrase 2	CAH2_HUMAN	2.919
1vj5	Epoxide hydrolase 2	HYES_HUMAN	2.919
2baq	Mitogen-activated protein kinase 14	Q16539	2.918
11gs	Glutathione S-transferase P	GSTP1_HUMAN	2.918
1oth	Ornithine carbamoyltransferase, mitochondrial	P00480	2.918
1h6g	Catenin alpha-1	CTNA1_HUMAN	2.917
1xlu	Cholinesterase	CHLE_HUMAN	2.917
1a4w	Prothrombin	THRB_HUMAN	2.916
2itz	Epidermal growth factor receptor	EGFR_HUMAN	2.916
3bej	Bile acid receptor	NR1H4_HUMAN	2.915
1bmq	Caspase-1	CASP1_HUMAN	2.915
1bx4	Adenosine kinase	ADK_HUMAN	2.914
2c5v	Cyclin-A2	CCNA2_HUMAN	2.914
1svg	cAMP-dependent protein kinase catalytic subunit alpha	P00517	2.911
1nhx	Phosphoenolpyruvate carboxykinase, cytosolic [GTP]	P35558	2.91
1w8c	Cell division protein kinase 2	P24941	2.91
1ym4	Beta-secretase 1	BACE1_HUMAN	2.909
1uu9	3-phosphoinositide-dependent protein kinase 1	PDPK1_HUMAN	2.909
1yvj	Tyrosine-protein kinase JAK3	JAK3_HUMAN	2.909
1pl1	Glutathione S-transferase A1	P08263	2.907
1veb	cAMP-dependent protein kinase catalytic subunit alpha	P00517	2.907
1lbk	Glutathione S-transferase P	GSTP1_HUMAN	2.907
1fkg	Peptidyl-prolyl cis-trans isomerase FKBP1A	FKB1A_HUMAN	2.906
2aeb	Arginase-1	ARGI1_HUMAN	2.906
2p4j	Beta-secretase 1	BACE1_HUMAN	2.905
1hna	Glutathione S-transferase Mu 2	GSTM2_HUMAN	2.905
1wbs	Mitogen-activated protein kinase 14	Q16539	2.905
3b2r	cGMP-specific 3,5-cyclic phosphodiesterase	PDE5A_HUMAN	2.904
1sve	cAMP-dependent protein kinase catalytic subunit alpha	P00517	2.904
1bnu	Carbonic anhydrase 2	CAH2_HUMAN	2.903
1mmb	Neutrophil collagenase	MMP8_HUMAN	2.901

1yxt	Proto-oncogene serine/threonine-protein kinase Pim-1	PIM1_HUMAN	2.901
2f3e	Beta-secretase 1	BACE1_HUMAN	2.9
1m7q	Mitogen-activated protein kinase 14	Q16539	2.9
2il2	Renin	RENI_HUMAN	2.899
1iz2	Alpha-1-antitrypsin	A1AT_HUMAN	2.899
1gni	Serum albumin	ALBU_HUMAN	2.898
2fjn	Tyrosine-protein phosphatase non-receptor type 1	PTN1_HUMAN	2.898
2ofu	Proto-oncogene tyrosine-protein kinase LCK	LCK_HUMAN	2.897
1q11	Tyrosyl-tRNA synthetase, cytoplasmic	P54577	2.897
1rs0	Complement factor B	CFAB_HUMAN	2.897
2obj	Proto-oncogene serine/threonine-protein kinase Pim-1	P11309	2.897
3d83	Mitogen-activated protein kinase 14	Q16539	2.896
2p4i	Angiopoietin-1 receptor	TIE2_HUMAN	2.896
3cpu	Pancreatic alpha-amylase	AMYP_HUMAN	2.896
1xbt	Thymidine kinase, cytosolic	P04183	2.896
1il0	Hydroxyacyl-coenzyme A dehydrogenase, mitochondrial	Q16836	2.896
1ove	Mitogen-activated protein kinase 14	Q16539	2.895
2gde	Prothrombin	THRB_HUMAN	2.895
1o4q	Proto-oncogene tyrosine-protein kinase Src	SRC_HUMAN	2.895
1svh	cAMP-dependent protein kinase, alpha-catalytic subunit	P00517	2.895
1yq7	Farnesyl pyrophosphate synthetase	FPPS_HUMAN	2.895
1hi4	Non-secretory ribonuclease	P10153	2.894
1n6i	Ras-related protein Rab-5A	RAB5A_HUMAN	2.893
1e4h	Transthyretin	TTHY_HUMAN	2.893
1kv2	Mitogen-activated protein kinase 14	Q16539	2.893
1xur	Collagenase 3	MMP13_HUMAN	2.893
1ezq	Coagulation factor X	FA10_HUMAN	2.892
1xs7	Beta-secretase 1	BACE1_HUMAN	2.892
1sz7	Trafficking protein particle complex subunit 3	O43617	2.892
1ivh	Isovaleryl-CoA dehydrogenase, mitochondrial	P26440	2.89
2b4y	NAD-dependent deacetylase sirtuin-5	SIRT5_HUMAN	2.89
2zb0	Mitogen-activated protein kinase 14	Q16539	2.89
1np0	Beta-hexosaminidase subunit beta	P07686	2.89
2uwo	Coagulation factor X	FA10_HUMAN	2.89
1hk3	Serum albumin	ALBU_HUMAN	2.89
1uej	Uridine-cytidine kinase 2	Q9BZX2	2.889
1gih	Cell division protein kinase 2	P24941	2.889
3h0y	NONE	NONE	2.888

Table S2. Potential Targets of Rhein after Accurate Molecular Docking

Targets Name	Gene Symbol	PDB-ID	Value of docking with rhein	Value of docking with experimental ligand
ABO blood group	ABO	1LZJ	-6	-4.9
		1R7U	-5.8	-4.5
		1R81	-5.7	-5.1
		1WT1	-6.1	-6
Alcohol dehydrogenase 5 (class III), chi polypeptide	ADH5	1M6W	-6.1	-4
Adenosine kinase	ADK	1BX4	-8	-7.4
Aldo-keto reductase family 1, member B1 (aldose reductase)	AKR1B1	1X98	-8.6	-7.5
Albumin	ALB	1GNI	-9.3	-6.7
		1GNJ	-8.5	-7.6
		1HK3	-8.7	-7.5
Amylase, alpha 1A (salivary)	AMY1A	1Q4N	-6.9	-4.6
Amylase, alpha 1B (salivary)	AMY1B			
Amylase, alpha 1C (salivary)	AMY1C			
Amylase, alpha 2A (pancreatic)	AMY2A	1XH0	-8.8	-7.6
		3CPU	-8	-5.6
Angiogenin, ribonuclease, RNase A family, 5	ANG	1B1I	-4.4	-3.5
Aurora kinase A	AURKA	3H0Y	-9.6	-9
Beta-site APP-cleaving enzyme 1	BACE1	1XS7	-8.5	-8.3
		2OAH	-9.2	-8.1
		2OF0	-6.7	-5.9
		3DUY	-8.6	-7.9
		3DV1	-8.7	-8
Butyrylcholinesterase	BCHE	1XLU	-4.8	-4.7
Betaine-homocysteine methyltransferase	BHMT	1LT8	-5.9	-5.7
Baculoviral IAP repeat-containing 7	BIRC7	3F7I	-6.6	-6.5
Carbonic anhydrase II	CA2	1BN3	-7.6	-6.6
		1BNQ	-7.7	-7
		1BNU	-8.1	-7.6
		1BNV	-7.5	-7.4
		2HOC	-7.9	-7
Carbonyl reductase 1	CBR1	1WMA	-6.7	-6.1
Cyclin A2	CCNA2	2C5V	-9.3	-8.9
		2IW6	-9.2	-7.8
Cyclin-dependent kinase 2	CDK2	1DI8	-9.8	-8.9
		1H01	-10.1	-8.8
		1H07	-9.6	-7.7

		1OIQ	-9.7	-7.8
		1OIR	-9.2	-8.7
		1V1K	-9.4	-8.7
		1W8C	-8.2	-7.5
		2C5V	-9.3	-8.9
		2C6I	-8.5	-8.2
		2IW6	-9.2	-7.8
Cyclin-dependent kinase 6	CDK6	1XO2	-10.8	-10.2
Cathepsin B	CTSB	1CSB	-7.4	-7
		1GMY	-7.3	-7.2
Death-associated protein kinase 1	DAPK1	1JKL	-9.1	-8.7
Dipeptidyl-peptidase 4	DPP4	1RWQ	-8.6	-6.8
Deoxythymidylate kinase (thymidylate kinase)	DTYMK	1E9A	-9.5	-8.7
Epidermal growth factor receptor	EGFR	2ITO	-9.2	-8.1
		2ITZ	-9	-8
Coagulation factor XI	F11	1ZPB	-7.2	-7.1
Coagulation factor II (thrombin)	F2	2GDE	-8.6	-7.8
Coagulation factor III (thromboplastin, tissue factor)	F3	1DAN	-8.1	-7.1
Coagulation factor VII (serum prothrombin conversion accelerator)	F7	1DAN	-8.1	-7.1
Fatty acid binding protein 3, muscle and heart (mammary-derived growth inhibitor)	FABP3	1HMS	-8.6	-5.9
Farnesyl diphosphate synthase (farnesyl pyrophosphate synthetase, dimethylallyltranstransferase, geranyltranstransferase)	FDPS	1YQ7	-8.3	-6.4
Fibrinogen gamma chain	FGG	1DUG	-5.3	-4.9
FK506 binding protein 1A, 12kDa	FKBP1A	1J4H	-7.7	-7.5
		1J4I	-6.2	-5.8
Glycogen synthase kinase 3 beta	GSK3B	1Q3D	-8.7	-8.4
		3F7Z	-8.8	-8.2
Glutathione reductase	GSR	1DNC	-6.1	-4.8
		1GRE	-6	-5.1
		1GSN	-6.3	-4.7
Glutathione S-transferase alpha 1	GSTA1	1GSF	-8.9	-7.1
		1GUH	-8.3	-7.7
		1LBK	-7.1	-5.7
		1PL1	-7.9	-7.2
Glutathione S-transferase mu 1	GSTM1	1YJ6	-6	-5.3
Glutathione S-transferase mu 2 (muscle)	GSTM2	1HNA	-6.6	-5.5
Glutathione S-transferase pi 1	GSTP1	11GS	-7.8	-5.7
		17GS	-7.9	-6.2

		1LBK	-7.1	-5.7
		1MD4	-7.1	-5.6
		9GSS	-7.8	-6
Histidine triad nucleotide binding protein 1	HINT1	1KPF	-8.3	-7.3
Heat shock protein 90kDa alpha (cytosolic), class A member 1	HSP90AA1	1UY6	-9.6	-7.9
		1UYG	-9	-8.2
		2WI5	-8.1	-7.5
		3BM9	-8	-7.8
Isovaleryl Coenzyme A dehydrogenase	IVD	1IVH	-7.9	-7.1
Janus kinase 3	JAK3	1YVJ	-8.7	-8.5
K(lysine) acetyltransferase 2B	KAT2B	1CM0	-8.7	-6.4
Lymphocyte-specific protein tyrosine kinase	LCK	1QPE	-8.3	-8.1
		2OFU	-8.6	-8.2
Monoamine oxidase B	MAOB	2BK3	-9.6	-7.6
Mitogen-activated protein kinase 10	MAPK10	3FV8	-10.2	-7.6
Mitogen-activated protein kinase 14	MAPK14	1DI9	-7.8	-6.4
		2BAQ	-9	-8.4
		2GFS	-9.1	-8.9
Mitogen-activated protein kinase 8	MAPK8	2NO3	-8	-7.3
Methionyl aminopeptidase 2	METAP2	1B59	-8.5	-7.1
		1B6A	-8.6	-7.6
		1BOA	-8.1	-5.9
Matrix metallopeptidase 3 (stromelysin 1, progelatinase)	MMP3	1BIW	-7.8	-6.7
Nicotinamide nucleotide adenylyltransferase 3	NMNAT3	1NUS	-8.5	-7.7
5',3'-nucleotidase, mitochondrial	NT5M	1Q91	-10.4	-10
Ornithine aminotransferase (gyrate atrophy)	OAT	2CAN	-8.3	-6.4
Phosphoenolpyruvate carboxykinase 1 (soluble)	PCK1	1M51	-9.3	-8.4
		1NHX	-10	-8.7
Phosphodiesterase 4B, cAMP-specific (phosphodiesterase E4 dunce homolog, Drosophila)	PDE4B	1RO9	-9.7	-7.4
Phosphodiesterase 4D, cAMP-specific (phosphodiesterase E3 dunce homolog, Drosophila)	PDE4D	1Y2E	-9.3	-7.4
		1Y2K	-9.3	-8.1
Phosphodiesterase 5A, cGMP-specific	PDE5A	3B2R	-9.9	-9.6
3-phosphoinositide dependent protein kinase-1	PDPK1	2PE1	-9.8	-9.2
Pim-1 oncogene	PIM1	1YXT	-9.7	-9.5
		2OBJ	-9.2	-9.1
Phospholipase A2, group IIA (platelets, synovial fluid)	PLA2G2A	1KQU	-8.7	-7.9
Protein tyrosine phosphatase, non-receptor	PTPN1	1JF7	-8.5	-8.4

type 1		1NWE	-7.7	-7.4
RAB5A, member RAS oncogene family	RAB5A	1N6I	-9.1	-9
Retinoic acid receptor, gamma	RARG	1FD0	-8.3	8.9
Serpin peptidase inhibitor, clade A (alpha-1 antiproteinase, antitrypsin), member 1	SERPINA1	1IZ2	-7.8	-6.2
Sirtuin (silent mating type information regulation 2 homolog) 5 (S. cerevisiae)	SIRT5	2B4Y	-6.8	-5.5
Sulfotransferase family 1E, estrogen-preferring, member 1	SULT1E1	1G3M	-8.8	-8.3

Table S3. Sorting results of topological parameters.

Target	Betweenness Centrality	Target	Degree	Target	Closeness Centrality	Target	Clustering Coefficient	Target	Topological Coefficient
HSP90AA1	4.74E-02	HSP90AA1	767	HSP90AA1	5.54E-01	IVD	1.00E+00	IVD	5.18E-01
EGFR	2.71E-02	CDK2	648	CDK2	5.33E-01	MAOB	6.67E-01	MAOB	3.46E-01
CDK2	1.96E-02	EGFR	551	EGFR	5.26E-01	GSTM2	6.67E-01	GSTM2	3.35E-01
ALB	1.65E-02	GSK3B	341	GSK3B	5.08E-01	DTYMK	6.00E-01	ADK	3.34E-01
GSK3B	1.32E-02	MAPK14	284	MAPK14	5.08E-01	PDE5A	5.00E-01	BHMT	3.00E-01
RELA	7.77E-03	RELA	258	RELA	4.95E-01	GSTM1	5.00E-01	PDE5A	2.69E-01
MAPK14	7.65E-03	NFKB1	213	NFKB1	4.95E-01	SIRT5	3.81E-01	GSTM1	2.68E-01
NFKB1	3.64E-03	KAT2B	178	MAPK8	4.86E-01	BIRC7	3.53E-01	DTYMK	2.60E-01
DPP4	3.48E-03	CASP3	172	CASP3	4.85E-01	F3	3.33E-01	AMY2A	2.58E-01
MAPK8	3.21E-03	MAPK8	166	LCK	4.84E-01	PDE4B	3.33E-01	SIRT5	2.25E-01
LCK	2.79E-03	ALB	164	KAT2B	4.81E-01	HINT1	2.48E-01	AMY1A	2.00E-01
CDK6	2.77E-03	LCK	142	ALB	4.80E-01	GSTP1	2.47E-01	AMY1B	2.00E-01
RAB5A	2.75E-03	CASP8	132	CASP8	4.80E-01	FDPS	2.43E-01	AMY1C	2.00E-01
SERPINA1	2.44E-03	RARA	130	RXRA	4.78E-01	ADH5	2.42E-01	PDE4B	1.92E-01
CTSB	2.41E-03	RXRA	127	CDK6	4.77E-01	GSR	2.31E-01	F3	1.76E-01
CASP3	2.38E-03	CDK6	109	RARA	4.76E-01	FGG	2.12E-01	GSTA1	1.45E-01
KAT2B	2.37E-03	AURKA	91	AURKA	4.76E-01	BHMT	2.00E-01	F11	1.34E-01
RARA	1.98E-03	CCNA2	80	PTPN1	4.74E-01	JAK3	1.89E-01	ANG	1.22E-01
VEGFA	1.78E-03	RAB5A	76	PDE4D	4.70E-01	F11	1.79E-01	SULT1E1	1.17E-01
CASP8	1.65E-03	PCK1	72	CCNA2	4.69E-01	DAPK1	1.71E-01	ADH5	1.13E-01
RXRA	1.63E-03	PTPN1	71	PIM1	4.69E-01	PDE4D	1.70E-01	FGG	1.10E-01
F2	1.52E-03	BAX	59	DAPK1	4.67E-01	FABP3	1.67E-01	F7	1.02E-01
GSTA1	1.20E-03	JAK3	47	PCK1	4.67E-01	CCNA2	1.62E-01	FDPS	1.02E-01

MMP3	7.69E-04	CTSB	44	MAPK10	4.67E-01	CBR1	1.58E-01	HINT1	9.82E-02
BACE1	7.28E-04	MAPK10	44	BAX	4.65E-01	METAP2	1.57E-01	FABP3	9.39E-02
PDPK1	6.92E-04	F2	43	CBR1	4.64E-01	BAX	1.36E-01	CBR1	9.15E-02
PLA2G2A	6.79E-04	GSTP1	43	GSTP1	4.64E-01	CA2	1.32E-01	OAT	9.13E-02
ADK	6.49E-04	PDPK1	40	CTSB	4.64E-01	PIM1	1.29E-01	AKR1B1	9.12E-02
DAPK1	6.45E-04	DAPK1	38	VEGFA	4.63E-01	BACE1	1.26E-01	GSTP1	8.79E-02
ANG	6.24E-04	SERPINA1	36	FKBP1A	4.62E-01	FKBP1A	1.22E-01	METAP2	8.79E-02
AURKA	6.08E-04	PIM1	36	ADH5	4.61E-01	PCK1	1.21E-01	BIRC7	8.50E-02
SULT1E1	6.06E-04	RARG	35	SIRT5	4.60E-01	PTPN1	1.16E-01	CA2	8.12E-02
BAX	5.51E-04	PDE4D	33	JAK3	4.60E-01	OAT	1.13E-01	PDE4D	8.02E-02
PTPN1	4.28E-04	DPP4	31	METAP2	4.60E-01	MAPK10	1.12E-01	MMP3	7.53E-02
RARG	4.20E-04	FKBP1A	30	RAB5A	4.60E-01	VEGFA	1.06E-01	LOX	7.23E-02
MAPK10	3.84E-04	CBR1	29	FDPS	4.58E-01	AKR1B1	1.03E-01	DAPK1	6.64E-02
CA2	3.82E-04	VEGFA	28	BACE1	4.57E-01	RXRA	1.03E-01	PIM1	6.58E-02
F7	3.53E-04	GSR	26	SERPINA1	4.57E-01	KAT2B	1.01E-01	GSR	6.19E-02
PCK1	3.00E-04	OAT	25	RARG	4.57E-01	RARG	9.75E-02	FKBP1A	6.15E-02
JAK3	2.68E-04	PLA2G2A	24	HINT1	4.56E-01	CASP8	9.40E-02	PLA2G2A	6.07E-02
GSR	2.27E-04	BACE1	23	OAT	4.56E-01	F7	9.09E-02	BACE1	5.84E-02
FGG	2.16E-04	METAP2	21	GSR	4.55E-01	RARA	8.81E-02	VEGFA	5.55E-02
CCNA2	1.90E-04	LOX	19	PDE4B	4.53E-01	LCK	8.47E-02	MAPK10	5.05E-02
PDE4D	1.39E-04	HINT1	18	AKR1B1	4.53E-01	AURKA	8.35E-02	PDPK1	4.62E-02
LOX	1.22E-04	BIRC7	18	BIRC7	4.52E-01	NFKB1	8.31E-02	PCK1	4.54E-02
AKR1B1	1.18E-04	MMP3	17	CA2	4.51E-01	PLA2G2A	7.25E-02	PTPN1	4.50E-02
FKBP1A	1.13E-04	CA2	17	DTYMK	4.51E-01	LOX	7.02E-02	CCNA2	4.45E-02
PIM1	1.05E-04	AKR1B1	17	FABP3	4.50E-01	MAPK8	6.81E-02	RARG	4.39E-02
FABP3	8.69E-05	FDPS	17	F3	4.50E-01	CTSB	6.45E-02	DPP4	4.38E-02

F11	8.02E-05	ADH5	14	PDE5A	4.48E-01	F2	6.20E-02	BAX	4.32E-02
F3	4.99E-05	SULT1E1	13	GSTM1	4.48E-01	RELA	6.04E-02	SERPINA1	4.09E-02
GSTP1	4.44E-05	FGG	12	MAOB	4.48E-01	SERPINA1	6.03E-02	JAK3	4.07E-02
METAP2	4.26E-05	FABP3	12	IVD	4.47E-01	CASP3	5.48E-02	CTSB	3.91E-02
CBR1	3.84E-05	ANG	11	ADK	4.47E-01	MAPK14	5.43E-02	AURKA	3.67E-02
HINT1	2.62E-05	F7	11	GSTM2	4.47E-01	CDK6	5.42E-02	F2	3.32E-02
ADH5	2.60E-05	GSTA1	8	PDPK1	3.69E-01	RAB5A	5.23E-02	CASP8	3.29E-02
OAT	2.48E-05	F11	8	LOX	3.69E-01	GSK3B	3.95E-02	KAT2B	3.22E-02
FDPS	2.25E-05	SIRT5	7	GSTA1	3.63E-01	SULT1E1	3.85E-02	NFKB1	3.21E-02
AMY2A	2.16E-05	F3	6	F2	3.62E-01	CDK2	3.80E-02	RARA	3.09E-02
PDE4B	1.22E-05	PDE4B	6	SULT1E1	3.57E-01	DPP4	3.66E-02	RXRA	3.00E-02
PDE5A	9.88E-06	AMY1A	6	FGG	3.51E-01	ANG	3.64E-02	MAPK8	2.92E-02
BIRC7	4.34E-06	AMY1B	6	F11	3.51E-01	GSTA1	3.57E-02	CDK6	2.81E-02
AMY1A	4.27E-06	AMY1C	6	ANG	3.51E-01	HSP90AA1	2.92E-02	LCK	2.78E-02
AMY1B	4.27E-06	DTYMK	5	PLA2G2A	3.50E-01	EGFR	2.60E-02	MAPK14	2.75E-02
AMY1C	4.27E-06	BHMT	5	F7	3.46E-01	MMP3	2.21E-02	CASP3	2.71E-02
SIRT5	3.94E-06	AMY2A	4	DPP4	3.41E-01	ALB	2.10E-02	RELA	2.69E-02
DTYMK	1.65E-06	PDE5A	4	MMP3	3.33E-01	PDPK1	1.92E-02	GSK3B	2.34E-02
BHMT	8.00E-07	GSTM1	4	AMY2A	3.32E-01	ADK	0.00E+00	RAB5A	2.26E-02
GSTM1	3.30E-07	ADK	3	AMY1A	3.23E-01	AMY2A	0.00E+00	CDK2	2.25E-02
MAOB	1.20E-07	MAOB	3	AMY1B	3.23E-01	AMY1A	0.00E+00	HSP90AA1	1.93E-02
GSTM2	1.00E-07	GSTM2	3	AMY1C	3.23E-01	AMY1B	0.00E+00	ALB	1.81E-02
IVD	0.00E+00	IVD	2	ABO	3.18E-01	AMY1C	0.00E+00	EGFR	1.76E-02
ABO	0.00E+00	ABO	1	BHMT	3.15E-01	ABO	0.00E+00	ABO	0.00E+00
BCHE	0.00E+00	BCHE	1	BCHE	3.09E-01	BCHE	0.00E+00	BCHE	0.00E+00
NMNAT3	0.00E+00	NMNAT3	0	NMNAT3	0.00E+00	NMNAT3	0.00E+00	NMNAT3	0.00E+00

NT5M	0.00E+00	NT5M	0	NT5M	0.00E+00	NT5M	0.00E+00	NT5M	0.00E+00
SRD5A2	0.00E+00	SRD5A2	0	SRD5A2	0.00E+00	SRD5A2	0.00E+00	SRD5A2	0.00E+00

Table S4. 15 Enriched Proteins in 11 KEGG Pathways

Term	Count	%	PValue	Genes
hsa04151:PI3K-Akt signaling pathway	9	42.85714	1.66E-06	EGFR, HSP90AA1, RELA, RXRA, GSK3B, VEGFA, NFKB1, CDK6, CDK2
hsa04668:TNF signaling pathway	6	28.57143	7.57E-06	CASP3, MAPK14, RELA, CASP8, NFKB1, MAPK8
hsa04014:Ras signaling pathway	6	28.57143	2.86E-04	EGFR, RELA, VEGFA, RAB5A, NFKB1, MAPK8
hsa04010:MAPK signaling pathway	6	28.57143	5.00E-04	EGFR, CASP3, MAPK14, RELA, NFKB1, MAPK8
hsa04210:Apoptosis	4	19.04762	6.02E-04	CASP3, RELA, CASP8, NFKB1
hsa04115:p53 signaling pathway	4	19.04762	7.56E-04	CASP3, CASP8, CDK6, CDK2
hsa04066:HIF-1 signaling pathway	4	19.04762	0.002273	EGFR, RELA, VEGFA, NFKB1
hsa04068:FoxO signaling pathway	4	19.04762	0.005504	EGFR, MAPK14, MAPK8, CDK2
hsa04064:NF-kappa B signaling pathway	3	14.28571	0.023322	RELA, LCK, NFKB1
hsa04012:ErbB signaling pathway	3	14.28571	0.023322	EGFR, GSK3B, MAPK8
hsa04110:Cell cycle	3	14.28571	0.044769	GSK3B, CDK6, CDK2

Fig S1. The positive and negative control signal for SPR

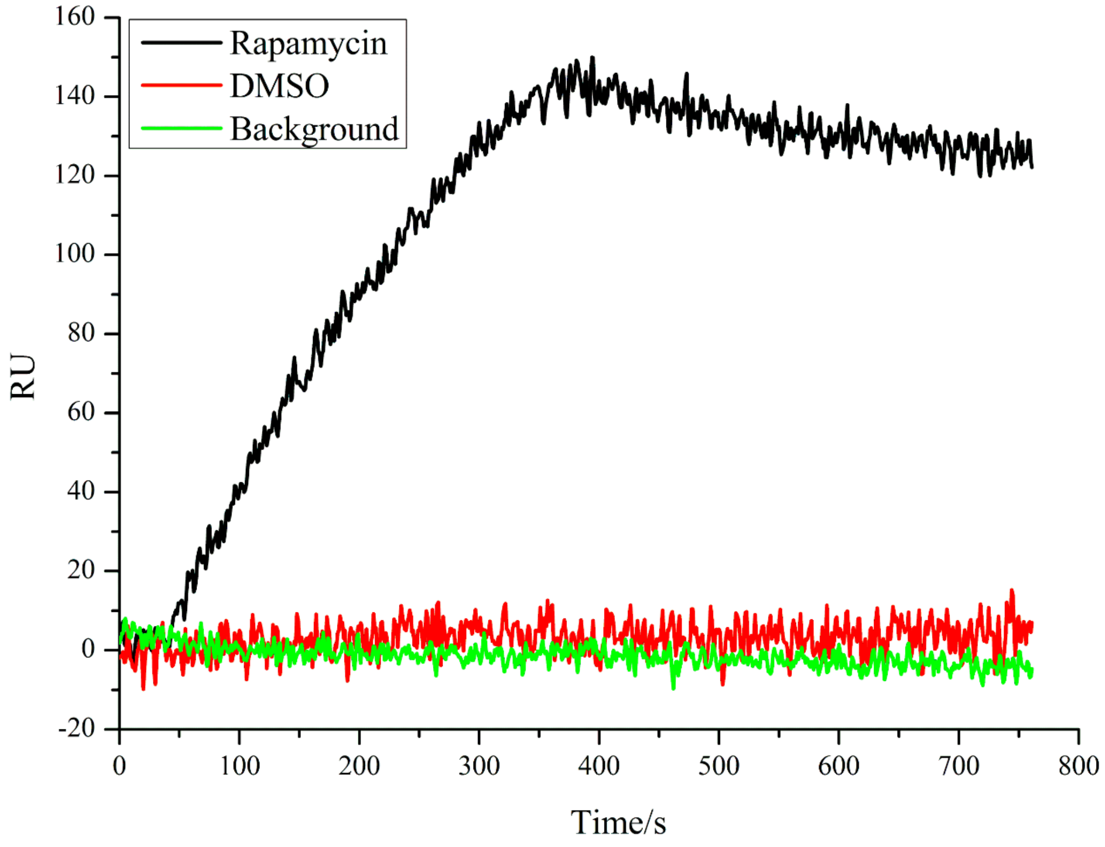

Supplement: Supplementary file 1 — Table S1. Inverse Docking Result. Table S2. Potential Targets of Rhein after Accurate Molecular Docking. Table S3. Sorting results of topological parameters. Table S4. 15 Enriched Proteins in 11 KEGG Pathways. Figure S1. The positive and negative control signal for SPR. (PDF 637 kb) [file 12859_2018_2346_MOESM1_ESM.pdf]
